# Transgender community resilience on YouTube: Constructing an informational, emotional, and sociorelational support exchange

**DOI:** 10.1002/jcop.22781

**Published:** 2021-12-23

**Authors:** Beatrice Rothbaum, Chana Etengoff, Elizabeth Uribe

**Affiliations:** ^1^ Derner School of Psychology Adelphi University Garden City New York USA

**Keywords:** community support, gender minorities, online social support, relational resilience, transgender, Transvlogs, YouTube

## Abstract

This strength‐based, mixed‐methods study explored how trans individuals utilize transvlogs as a community building and resilience resource. Eighty‐six transvlog viewers explained their motivation for viewing transvlogs and additionally rated their self‐efficacy and well‐being. Narrative analyses indicate that participants viewed transvlogs to gain informational, emotional, and sociorelational resources. Twelve percent of participants additionally shared those resources with others and contributed to a relational resilience exchange. In addition, transvlog viewers' self‐efficacy and well‐being scores were higher than previously reported means. While prior research has noted the benefits of transvlog creation, the present study furthers this study by suggesting that transvlog viewers interactionally benefit as well. Participants in this study were not passive viewers, but rather active, agentive contributors to a trans community resilience exchange. Building on this study, we conclude with a discussion of how therapists can incorporate extant trans‐created resources to foster trans community resilience.

## 1 INTRODUCTION

Many trans^1^ individuals endure pervasive stigma, prejudice, and discrimination related to their gender identity, often contributing to chronic psychological distress (W. O. Bockting et al., 2013). For example, trans adults experience elevated rates of suicidality, depression, anxiety, substance use, and trauma compared with cisgender^2^ adults (Budge et al., 2013; Valentine & Shipherd, 2018). Alternatively, social support from family, friends, and the trans community may buffer the risk of negative mental health outcomes (W. O. Bockting et al., 2013; Budge et al., 2013; Pflum et al., 2015; Valentine & Shipherd, 2018). However, family members often withhold support and friends may commit painful microaggressions, positioning trans community support as a primary source of social support for trans individuals (Factor & Rothblum, 2007; Galupo et al., 2014; Pinto et al., 2008). For trans individuals facing social rejection and discrimination, trans community engagement, both online and offline, can promote feelings of visibility, belonging, inspiration, and self‐acceptance (Paceley et al., 2020; Puckett et al., 2019). Although several studies have analyzed the benefits of trans individuals *creating* online video blogs (Etengoff, 2019; Horak, 2014; Raun,2015a, 2015b), there is a dearth of research exploring trans video blog *viewers'* experiences. However, trans online video blogs, otherwise known as transvlogs, are inherently interactional, as viewers and creators dialog via comment forums and video responses (Etengoff & Rodriguez, 2016; Etengoff, 2019; Rotman & Preece, 2010). Therefore, this study focuses on how trans individuals utilize transvlogs as a community support exchange to mediate the challenges of systemic and interpersonal oppression.

### 1.1 Transvlogs, transvloggers, and transvlog viewers

Online networks provide forums for trans individuals to continuously construct and edit their online personas, find gender transition information, organize activism efforts, network, and build community (Etengoff & Rodriguez, 2016; Etengoff, 2019; Gauthier & Chaudoir, 2004; McInroy & Craig, 2015; Shapiro, 2004; Whittle, 1998). For example, transvloggers self‐produce and publicly upload their transition stories to online video platforms such as YouTube (Etengoff & Rodriguez, 2016; Raun, 2010). Transvlog viewers can then reference these serial video blogs as step‐by‐step guides on coming out, hormone dosages, gender affirmation surgery, and gender presentation (Kosenko et al., 2018; Miller, 2017).

Transvlogs often function as transition diaries, allowing transvloggers to express gender on their own terms, update viewers on physical changes, and share emotional journeys (Miller, 2017; Raun, 2015b). Transvloggers may also explain trans‐relevant terms and skills such as gendered voice modulation or chest binding^3^ (Horak, 2014). Many transvloggers also discuss their experiences with dating, depression, identity disclosure, family interactions, bullying, and discrimination in a way that is often absent from mainstream media's sensationalization of trans lives (Etengoff, 2019; Jenzen, 2017; Miller, 2017). Extending this study, Etengoff (2019) reported that many transvloggers additionally use the platform to educate their audience, disrupt transprejudice, change gender‐binary norms, and inspire community action.

Moreover, for those who cannot easily access a local trans community (e.g., rural communities and religious communities), the internet often plays a vital role in trans identity and community development (Gray, 2009; Shapiro, 2004; Singh, 2013). Trans individuals have reported using the relative safety of virtual communities to disclose their gender identities with decreased stigma and victimization (Yokotani & Takano, 2021). Although, we do acknowledge that trans‐related cyber bullying does occur and can negatively impact trans mental health as well (Abreu & Kenny, 2018). Yet despite the risk of cyberbullying, trans individuals have reported engaging social media (e.g., Facebook) as a therapeutic tool for building community by sharing transition‐related experiences, developing close relationships, seeking and offering support, and having a mutually positive impact on others (Cipolletta et al., 2017; Hawkins & Haimson, 2018). Building on this foundation, the present article explores how transvlogs facilitate and support relational resilience pathways for the trans community.

### 1.2 Relational resilience

Emerging from the interaction between risk factors and protective mechanisms, *resilience* allows individuals to sustain positive development across behavioral, cognitive, and emotional domains despite significant stress and adversity (Rutter, 1987). Traditionally, psychologists have approached resilience as a component of internal personality characteristics, such as temperament and self‐esteem (Hartling, 2005; Singh & McKleroy, 2011). However, this focus on internal factors may undermine the pivotal role of external social factors, such as stigma or community support, for historically marginalized groups such as the trans community (Hartling, 2005; Singh & McKleroy, 2011). For example, research suggests that stigmatized individuals utilize *and* contribute to communities which provide a platform for resisting societal discrimination (Singh & McKleroy, 2011; Wexler et al., 2009). In this vein, Ungar (2008) frames resilience as:

Both the capacity of individuals to *navigate* their way to health‐sustaining resources, including opportunities to experience feelings of well‐being, and a condition of the individual's family, community and culture to provide these health resources and experiences in culturally meaningful ways (p. 225).

However, despite the inherently interactive construct of social support, many psychologists have traditionally studied social support as a unidirectional tool in which an individual receives support from others (often, more competent or privileged others) (Hartling, 2005). Contrastingly, Hartling (2005) positions relational resilience as an interactive, *bidirectional* exchange of support. Relational resilience proposes that individuals grow even amidst adversity and trauma via reciprocally engaging social relationships (Hartling, 2005; Walsh, 2003). For example, Howell et al. (2020) reported that relational resilience successfully mediated the association between adverse childhood experiences and adult depression. As Walsh (2003) explains, “resilience involves transcendence: from personal tragedy and suffering to concern and action on behalf of others, to prevent similar suffering” (p. 56). Self‐empowerment is attained through *mutual* empowerment; individuals can choose to strengthen their own resilience by contributing to the resilience of others (Hartling, 2005). Relational resilience is an inherently agentive theory, as even the most vulnerable of individuals can be active agents of their own collaborative and transformative development (see Stetsenko, 2017 for further discussion of transformative development theory). Supporting this stance, emerging research suggests that trans individuals may strengthen their own resilience by contributing to trans community resilience (Singh et al., 2011).

### 1.3 Trans resilience and online communities

Trans mental health research has traditionally focused on psychopathology and sampled participants from medical environments such as gender clinics or sexual health centers (Pitts et al., 2009). However, Burdge (2007) argues that trans affirmative research should also include an assessment of skills such as courage, resilience, authenticity, and social justice. To this end, this study builds on positive frameworks for trans development and growth by approaching transvlogs as a relational and community‐building tool for resilience.

Extant research on trans resilience has identified social support and community connectedness as protective factors against the negative effects of societal discrimination (Amodeo et al., 2018; Bowling et al., 2020; Breslow et al., 2015; Gonzalez et al., 2012; Meyer, 2015; Paceley et al., 2020; Pflum et al., 2015; Singh et al., 2011; Singh, 2013; Testa et al., 2014; Zeeman et al., 2017). Trans individuals can proactively decrease psychological distress by engaging in communal resilience strategies such as contributing to a trans community via social activism and serving as a positive role model for others (Gonzalez et al., 2012; Sánchez & Vilain, 2009; Singh et al., 2011, 2014). Social support from similar peers may normalize and validate emotional experiences related to trans discrimination (Pflum et al., 2015; Singh, 2013). For example, a recent study by Bowling et al. (2020) observed that trans group connection provided an opportunity to share personal stories and resilience strategies, leading to a sense of normalization and relief.

Relatedly, Singh et al. (2011) found that when under stress, trans individuals sought connections with peers for information on how to negotiate discrimination and how to access necessary care and resources. Furthermore, a study by Amodeo et al. (2018) investigating resilience in a group of eight Italian trans youths found that the process of *mirroring*—recognizing oneself in another who shares the same identity categories—increases resilience by reinforcing trans identity. When one group member found their experience reflected in another group member, they became more likely to share their own experiences of distress and were better at utilizing common coping strategies (Amodeo et al., 2018). In sum, trans peer groups create a sense of trust and closeness by providing a safe space to socialize, make sense of challenging events, and promote feelings of personal strength and stability (Amodeo et al., 2018; Zeeman et al., 2017).

Research suggests that social media use (e.g., Facebook, Twitter, and YouTube) can similarly empower trans individuals to feel positively about their (multiple) identities by normalizing their experiences and highlighting their strengths (Cannon et al., 2017; Singh, 2013; Singh et al., 2011). For example, transvloggers' autobiographical accounts of transdiscrimination, prejudice, and microaggressions can help trans viewers identify hostility in their own lives and learn about resilience/coping strategies (Etengoff, 2019; Miller, 2017; Singh, 2013). As trans individuals deepen their awareness of gender‐based oppression, they can more effectively negotiate interpersonal discrimination and more consciously refrain from internalizing negative societal messages, thereby decreasing their psychological distress (Breslow et al., 2015; Singh & McKleroy, 2011; Singh et al., 2011).

Craig et al. (2015) found that online social media interactions may increase resilience for trans youth by promoting relational skills that they can utilize during offline encounters, such as responding to positive and negative interpersonal feedback. By commenting on and creating content in response to experiences of discrimination on social media, trans youth are adaptively engaging in problem solving and resource management (Craig et al., 2015). Similarly, Singh (2013) found that social media empowered trans youth of color to embrace their intersectional identities and learn new strategies for addressing racism and trans discrimination in their offline lives. Building on these previous studies, the present article further explores the relational dynamics of trans resilience by examining transvlogs as a unique tool for exchanging bidirectional support and building trans community.

### 1.4 The present study

The present exploratory, mixed‐method study is part of a larger research project aimed at understanding self‐efficacy and well‐being in trans Americans. This specific segment of the study focused on how transvlog viewers use YouTube to build trans community resilience by addressing the following questions:

1. How do trans individuals describe their use of transvlogs?
2. How do transvlog viewers use the YouTube network to both gain from *and* give back to the online trans community? What resilience resources do transvlog viewers exchange with transvlog creators?
3. Do transvlog viewers report positive mental health scores, specifically, for well‐being and self‐efficacy?

## 2 METHOD

### 2.1 Participants

Eighty‐six participants (74% of recruited participants) reported engaging transvlogs and are included in this study. Participants were predominantly White (*N* = 73, 85%) and had an average age of 44 (range: 18–86; *SD* = 17.84 years). While all participants identified broadly as trans, participants reported a wide range of gender identities. For example, in some form, 27% identified as a transwoman (i.e., transfeminine, transgirl), 24% identified as a transman (i.e., transmasculine,transboy), 18% identified as nonbinary (i.e., genderfluid, agender), 14% currently identified as female (i.e., woman, femme), 11% identified as trans (i.e., transgender) with no additional gender qualifier, and 2% identified as male (i.e., boy). Participants' sexual orientation responses were similarly diverse. For example, 20% identified as bisexual, 16% as heterosexual, 15% as some other sexual orientation (i.e., demisexual, questioning), 14% as queer, 13% as lesbian, 12% as pansexual, and 7% as asexual. Participants were highly educated with 59% of participants having obtained a college degree or higher. However, income status was more diverse, with 41% earning less than $25,000 a year, 17% earning between $50,000 and $74,999, and 13% earning between $25,000  and $34,999. Income variation may be due to 18% of participants identifying as retired and 11% as unemployed.

### 2.2 Measures

The online survey began with three close‐ended and five open‐ended demographic questions (e.g., sexual orientation and gender identification). The narrative analysis portion of this study focused upon participants' responses to two open‐ended transvlog questions: (1) Why did you watch transvlogs? and (2) Did you find the transvlogs to be helpful? The survey continued with self‐efficacy and well‐being measures as described below.

*General self‐efficacy* was assessed with the *General Self‐Efficacy Scale* (GSE, Schwarzer & Jerusalem, 1995). Participants were asked to rate their agreement with 10 statements referring to successful coping (e.g., I can always manage to solve difficult problems if I try hard enough) using a 4‐point scale (1 = *Not at all true* to 4 =  *Exactly true*). Ratings were then summed to yield the final composite score with a range from 10 to 40, with higher scores indicating greater self‐efficacy. Reliability analyses indicate high consistency in participants' responses across items (Cronbach's *α* = 0.89).

*Mental well‐being* was assessed with the *Short Warwick‐Edinburgh Mental Well‐Being Scale* (SWEMWBS, Stewart‐Brown et al., 2009). Participants rated their agreement with seven positively worded mental health statements (e.g., I've been optimistic about the future; I've been feeling close to other people) using a 5‐point scale (1 = *None of the time* to 5 = *All of the time*). Response ratings were summed to yield a final composite score of 7–35, with greater scores indicating higher levels of mental well‐being. Reliability analyses indicate high consistency in participants' responses across items with a Cronbach's *α* of 0.84.

### 2.3 Recruitment and procedure

After obtaining institutional review board (IRB) approval, participants were recruited via trans online public forums, blogs, and listservs (e.g., Facebook pages, Twitter, and Reddit) as well as via flyers at trans and/or lesbian, gay, bisexual, transgender, and queer (LGBTQ) centers (e.g., college and local community centers). Survey inclusion criteria required that participants (a) identify as trans, (b) be above 18 years of age, and (c) currently reside in the United States. The survey was accessed online and hosted by Qualtrics, an online survey platform. Before beginning the survey, participants completed an informed consent form noting that participation was voluntary and there would be no compensation for participation. The survey did not ask participants for their names or emails and IP addresses were not collected.

### 2.4 Analysis procedure

Narrative analysis was chosen as this method uniquely allows individuals to recount their own lived experiences (Creswell & Poth, 2016). Building on this stance, we employed a transformative narrative framework that centers marginalized individuals as active agents of their own collaborative development (Stetsenko, 2017). The narrative analysis took into account not only individuals' lived experiences but also the social, cultural, and institutional context in which the individuals' experiences were constituted and enacted (Clandinin, 2013). Codes were conceptualized based on Hartling's (2005) and Walsh's (2003) interactive perspectives on relational resilience as a bidirectional exchange of support and mutual empowerment.

Narrative analysis began with the first author reading participants' responses for emergent themes and recurring patterns. The first author then created an initial list of four codes. After the second author reviewed the initial code list, both authors agreed on collapsing the categories into three primary motivations for watching vlogs: (1) emotional, (2) sociorelational, and (3) informational resources. Given that relational resilience includes both gaining and giving support to others, the authors created two subcategory codes to identify when participants (1) gained or (2) gave resources. The first author then reread the data to ensure goodness of fit. The final six codes are: emotional gaining, emotional giving, sociorelational gaining, sociorelational giving, informational gaining, and informational giving. The authors developed a codebook with definitions and examples for each of the six codes to establish inter‐rater reliability with the third author, an advanced clinical psychology doctoral student (i.e., secondary coder). The first author created 12 test narratives solely for inter‐rater training. The secondary coder was trained via Zoom, an online video conferencing platform, and was required to achieve a 90% rate of agreement with the first author on the test narratives. Test narrative disagreements were discussed before the secondary coder was given the full data set for coding. Initial inter‐coder reliability was 91% for the final data set. Discussion with the second author resolved the remaining number of dispute codes. To provide both depth and breadth, both qualitative and frequency analyses are reported (Wilkinson, 2000). Individual narratives were selected for inclusion in the manuscript if they were able to succinctly and comprehensively illustrate a category of relational resilience. In addition, we conducted exploratory quantitative data analyses to provide additional insight into participants' narratives as well as to better assess the overarching research questions. Due to the small sample size, by quantitative standards, we limited ourselves to descriptive data analyses and basic interstudy comparisons of participants' self‐efficacy and well‐being scores.

## 3 RESULTS

### 3.1 Emotional, sociorelational, and informational resources

Narrative analyses indicate that 85 participants (99%)^4^ watched transvlogs to gain informational, emotional, and sociorelational resources (see Table 1: resource types, frequencies, and sample narratives). Sixty‐four participants (74%) viewed transvlogs to access more than one resource (e.g., “helped me learn more about my emerging identity (code: informational) and connect with others (code: sociorelational)”). A smaller segment of viewers (12%) additionally cultivated relational resilience by *giving* those resources to others (See Table 1: resource types, frequencies, and sample narratives). Of the 10 participants that engaged transvlogs as relational resilience resources, nine shared multiple resources with others.

**Table 1:** Resource types, frequencies, and sample narratives

Type of resource	Gaining frequency *N* = 86	Giving frequency *N* = 86	Gaining sample narrative	Giving sample narrative
Informational	*n* = 59 (69%)	*n* = 5 (6%)	“These [YouTube] formats helped [me] to learn expressive skills such as tucking, wardrobe, and skin care” (52, “Gender fluid,” “Caucasian”)	“To collect teaching tools for the college courses I teach” (34, “nonbinary trans‐masculine,” “European‐American deconstructing whiteness”)
			“To see more diverse perspectives on transness” (31, “nonbinary boy,” “black/mixed”)	“To provide… information for myself and others” (18, “Male (Ftm),” “Caucasian”)
Emotional	*n* = 54 (63%)	*n* = 9 (10%)	“To… [learn] coping mechanisms to help deal with my transition. Yes, they give me hope that things can change for me in the future.” (31, “Female,” “Mixed White/Native American”)	“To learn [about] other people's experience AND, via [my] commentary, to add support.” (72, “female,” “White”)
			“…Hearing others tell their stories and experiences made me feel like I wasn't alone and wasn't a problem.” (27, “Transgender male,” “Halfrican American (half black and half white)”)	“To help other trans people coming to terms with their identity.” (19, “transman,” “White”)
Sociorelational	*n* = 61 (71%)	*n* = 8 (9%)	“To find people to relate to…” (34, “genderqueer androgyne,” “white Hispanic”)	“[I] Support by listening to others stories [on YouTube]” (68, “gender fluid,” “white”)
			“Now I watch for… social connection.”	“I may see opportunities to support others (if they ask for support) [on YouTube]” (64, “human gender,” “human race”)
			(27, “Transgender male,” “Halfrican American (half black and half white)”)	

*Note*: Narrative data were drawn from two open‐ended questions: (1) why did you watch transvlogs, and (2) did you find the transvlogs to be helpful?

#### 3.1.1 Informational resources

Fifty‐nine participants (69%) watched transvlogs to learn about the transition process and experience (e.g., “hormone treatment,” “terminology,” “product reviews,” “technical instruction on voice or appearance,” “physiological effects and inner emotions to transition steps”). For example, a 20‐year‐old “White/mixed race” transman from Montana, who described his sexual orientation as “complicated,” explained that he “learned a lot about the transition process and how to safely bind my chest [from viewing transvlogs].”

Of those who viewed transvlogs to gain informational resources, five (9%) additionally sought to share those resources with others (e.g., “family,” “my students,” “my [trans] groups”). One participant shared informational resources with other members of the trans community and three participants shared resources with those outside the trans community^5^. For example, a 34‐year‐old queer, “Euro‐American deconstructing whiteness” from Virginia, who described themselves as nonbinary and “trans‐masculine,” stated that they initially became a transvlog viewer to:

learn more about [the] medical transition, to learn more about other people's lived trans experience, to feel less isolated, to learn more about nonbinary identities, to collect teaching tools for the college courses I teach. I did find them [transvlogs] helpful. There's a lot of valuable information out there, they made me feel less alone, and they help me humanize trans people for my students.

In this quote, the participant explains that a notable part of their journey of self‐discovery involved using their knowledge acquired from transvlogs to benefit their college students (e.g., “…they help me humanize trans people for my students”) as well as themselves (e.g., “…to feel less isolated, to learn more about nonbinary identities”).

#### 3.1.2 Self‐Development and emotional resources

Fifty‐four participants (63%) shared that they watched transvlogs to gain emotional tools such as advice on how to regulate emotions (e.g., “they help my inner peace”), ways to improve self‐esteem and self‐acceptance (e.g., “they helped me accept who I am”), and pathways to foster hope for the post‐transition future (e.g., “that there was a real life after transitioning, that there was love after transitioning”). For example, a 27‐year‐old asexual, “Halfrican American (half black and half white)” transman from Tennessee shared that he used transvlogs to gain self‐compassion (e.g., “[transvlogs] made me feel like I wasn't a problem”) and to manage his expectations for the transition experience (e.g., “I also wanted to be prepared for the potential financial, social, and mental strains that I might experience”).

Of the participants who watched transvlogs to access emotional resources, nine (17%) stated that they actively shared those resources with others (e.g., “family,” “trans vloggers/artists,” “other trans people”). Seven participants shared emotional resources with fellow members of the trans community and two shared resources with those outside the trans community. For example, a 19‐year‐old pansexual, White transman from Arizona described how he first used transvlogs to better understand his own trans identity and emotions. He later used transvlogs to help other trans individuals strengthen their self‐knowledge and self‐acceptance. In his words,

I watched them [transvlogs] as I was coming to terms with my trans identity (approximately 6‐7 years ago) to better understand my feelings and experiences, and what the path to transition might look like…They [transvlogs] helped me understand myself and my emotions better. I have also used them to help other trans people coming to terms with their identity.

In the above quote, the participant describes how transvlogs helped him process his emotions (e.g., “…to better understand my feelings and experiences”) and gain clarity (e.g., “…what the path to transition might look like”). After actively acquiring the emotional resources he needed to come to terms with his trans identity, he was better able to help other trans people cope with similar struggles (e.g., “…used them to help other trans people coming to terms with their identity”).

#### 3.1.3 Sociorelational and community resources

Sixty‐one participants (71%) stated that they viewed transvlogs to gain sociorelational tools such as empathy for others (e.g., “it was the first time I saw and listened to anyone who was like me”), connecting with a trans community (e.g., “transvlogs give me a sense of community, especially given that I don't have too many transfolks in my life”), and navigating complex social environments (e.g., “[transvlogs] [help] me conceptualize trans spaces and the trans or cis[gender] people I might meet there so I'm more prepared to protect myself and engage people and discourses more carefully”).

Of those who watched transvlogs for sociorelational resources, eight participants (13%) also actively shared those tools with others (e.g., “my students,” “my [trans] groups”). Three participants described sharing relational resources with people outside the trans community (e.g., “how to make this [the transition process] easier on my family”) and five participants shared sociorelational resources with other members of the trans community. For example, a 68‐year‐old, White, lesbian transwoman from South Carolina watched transvlogs, “to see what others are saying or doing in other areas of the LGBT community and in other states [and] other countries. I learn[ed] info[rmation] or [transvlogs] reminded [me] of info[rmation] that I can carry back to my [trans] groups to keep them informed…”). Another participant, a 46‐year‐old White “genderqueer‐transmasculine” individual from Massachusetts who described their sexual orientation as “attracted to men,” stated that they viewed transvlogs to make their Episcopal church more understanding and welcoming for other trans folks. They explained,

For the church class I taught, I watched some [transvlog] episodes of Austen Hartke's ‘Transgender and Christian' and created a curriculum about it…Hartke's [transvlog] series gave our group some good ideas about how to make the church more trans‐inclusive.

In this narrative, the participant illustrates their process of gleaning important resources from an online trans community to strengthen and diversify their in‐person church community (e.g., “…gave our group some good ideas about how to make the church more trans‐inclusive”).

### 3.2 Transvlog engagement and mental health

Participants' average GSE (Schwarzer & Jerusalem, 1995) score was 30.88 (*SD* = 5.34), which is significantly higher than Schwarzer and Jerusalem's (1995) initial sample of cisgender male and female adults^6^ (*M* = 29.48, *SD* = 5.13), *t*(81) = 2.372, *p* = 0.02. Participants' average SWEMWBS (Stewart‐Brown et al., 2009) score of 23.98 (*SD* = 4.64) was significantly higher than previously sampled trans populations (Alanko & Lund, 2020) (*M* = 21.6, *SD* = 4.9), *t*(84) = 4.723, *p* = 0.00, although similar to Fat et al. (2017) initial sample of cisgender men (*M* = 23.67, *SD* = 3.92) and women (*M* = 23.59, *SD* = 3.87). Participants' average GSE and SWEMWBS scores were positively correlated, *r*(80) = 0.67, *p* < 0.001.

## 4 DISCUSSION

Drawing on a strength‐based research stance, the present article utilized a mixed‐methods approach to explore how 86 trans individuals used transvlogs as an interactive resilience and community building tool. Ninety‐nine percent of participants in this study accessed transvlogs as an informational, emotional, or sociorelational resource. In addition, 12% of participants shared these resources with others and agentively contributed to a dynamic relational resilience exchange. The comparatively low rates of participants' relational resilience engagement suggests that relational resilience may need to be supported via psychotherapy or participatory action research. Quantitative analyses indicate that participants in this study reported higher self‐efficacy and well‐being scores than previously sampled trans and cisgender populations, suggesting that transvlog engagement is an important pathway for trans resilience. Gonzalez et al. (2012) reported that a combination of agentic traits (e.g., self‐assertiveness and self‐confidence) and communal traits (e.g., empathy and cooperation) are associated with lower levels of depression and higher levels of resilience among transwomen. This study builds on this study by arguing that transvlog tools can support this dual resilience process. Though a larger sample size and pre‐ and postdata are required to unpack causal and mediational pathways, the current study's exploratory and descriptive analyses construct a strong theoretical foundation for understanding the role that online transvlog communities can play in supporting trans resilience.

This study contributes to previous work on the resilience benefits of online trans communities (i.e., Craig et al., 2015; Etengoff, 2019; Singh, 2013) by expanding the scope of psychological inquiry from online media content creators to include online media viewers. Given Craig et al.'s (2015) stance that LGBTQ + individuals' choice to engage online media is an act of resilience, proactive coping, and resource management, this study approached transvlog viewer engagement as a gateway to self and relational resilience pathways. Though prior work by Etengoff (2019) highlighted the transformative developmental processes of transvlog content creation, there is a lack of research focused on how transvlog viewers utilize these resources to support their transition experiences. This study's findings represent one of the first psychological inquiries into how trans viewers utilize transvlogs to gain and share informational, emotional, and sociorelational resources. These findings echo Selkie et al.'s (2020) recent qualitative study exploring how trans adolescents (15–18 years) use social media (e.g., YouTube, Instagram, Facebook, Twitter, Tumblr) to gain emotional support (e.g., hope for the future, decreased feelings of isolation), appraisal support (e.g., validation from peers) and informational support (e.g., tips for obtaining surgery) (Selkie et al., 2020). Moreover, this study is one of the first to suggest that online interactions may be a beneficial resource for trans adults (18–86 years) as well.

Many of the community‐based resilience pathways reported in this study mirror extant research findings with trans populations. For example, research indicates that the internet (e.g., websites, listservs, and online forums) can provide trans individuals from more isolated areas with a vibrant trans community to facilitate the building of a collective trans identity, the distribution of trans‐related information, and community organizing (Shapiro, 2004). Similarly, a recent study by Krueger and Young (2015) reported that trans individuals engage Twitter as a place for “camaraderie and support” from the trans community. In addition, a growing body of research has examined how trans viewers use YouTube as an educational tool to learn about gender identity, transitioning and how to combat stigma (Miller, 2017; Shapiro, 2004). Increasingly, trans individuals use YouTube as a psychological space in which to support, inform, and collaborate with others in the trans community (O'Neill, 2014). The strength‐based analysis framework in the present study reinforces previous research findings that trans individuals actively engage online media with a specific goal in mind (e.g., making sense of gender identity, building community; Kosenko et al., 2018). This study contributes to the larger positive psychology literature on trans individuals by expanding upon how trans individuals build resilience by seeking positive connections and support within their own online communities (McCullough et al., 2017).

### 4.1 Limitations

Findings from this study should be interpreted in light of several limitations. Below, we outline the primary limitations related to sampling and measurement.

#### 4.1.1 Sampling

Though this study's sample size of 86 is one of the largest qualitative samples of this population to date, it is a limited sample by quantitative standards and only descriptive analyses and basic mean comparisons were conducted. In addition, as with most sexual and gender minority research, the current sample represents only those participants who were interested and willing to participate (Gonsiorek & Weinrich, 1991). Moreover, this study's sample is reflective of the larger population's rates of transvlog use and as such, we were unable to recruit a large comparative sample of nonvlog viewers. Future researchers may therefore benefit from utilizing an experimental research design, which randomly assigns participants to various transvlog engagement groups. This study's generalizability is additionally limited by the disproportionate number of middle aged (*M* = 44 years; *SD* = 17.84 years), White (85%) and highly educated (e.g., 59% had a college degree or higher) participants. Future research should take additional steps to recruit and establish trust with trans people of color, who may be difficult for researchers to access due to their multiple marginalized identities and the associated sociocultural stigma (Wheeler, 2003).

#### 4.1.2 Method and measurement

As a result of the online presentation of the survey, participation was contingent on internet access. This is an important consideration, as internet access in the United States is mitigated by social class and race (Shapiro, 2004). Thus, lower participation rates for individuals from lower socioeconomic brackets and individuals of color may be explained by limited internet access. As with all online surveys, there may have been a potential for false responses. However, the lack of compensation for this study likely reduced the incentive for fraudulent behavior (Teitcher et al., 2015). The potential for bots was also decreased by alternating between quantitative and qualitative questions throughout the survey. In addition, while the online distribution of the survey may have supported a broader recruitment reach, the online method led to a structured qualitative design. Thus, participants could not engage the researchers to ask them to clarify their questions and researchers could not follow‐up with participants to ask them to explain their responses. Mental health benefits were assessed in terms of self‐efficacy and well‐being. However, there are many other possible mental health benefits, and the study could have been strengthened with additional measures such as general coping, interpersonal skills, and locus of control. Furthermore, this study reported mental health findings as compared with previously published population norms. While this is standard practice for clinical assessments and trans research (e.g., Etengoff & Rodriguez, 2020), future researchers may want to further explore potential differences between transvlog exposure/engagement groups utilizing a controlled experimental design.

### 4.2 Future directions

In addition to the above suggestions, future research would benefit from examining how trans individuals' online resilience strategies differ based on race, social class, sexual orientation, age, disability, and other intersectional factors. For example, Singh (2013) found that trans youth of color employ unique resilience strategies for recognizing and navigating racism and transprejudice, including racial/ethnic identity development, self‐advocacy, and connecting with other trans individuals of color. Additionally, research with black trans individuals has indicated that both their connection to members of their racial community and culturally specific coping skills facilitate resilience in multiple hostile contexts (Follins et al., 2014). Furthermore, Bariola et al. (2015) research with a national sample of trans Australians demonstrated that high income status and identifying as a heterosexual trans person are associated with greater resilience. Income may be a particularly salient factor for trans populations due to their disproportionately high rates of unemployment and income inequality (James et al., 2016). Expanding our contextual understanding of race, sexual identity, class, and social oppression is a prerequisite to creating more meaningful research designs and developing stronger partnerships between researchers and the trans community (Wheeler, 2003).

### 4.3 Clinical implications

Online resources have become an essential source of support for LGBTQ individuals during the COVID‐19 pandemic due to social distancing, sheltering in place with potentially unsupportive family or roommates, restricted access to LGBTQ community centers, reduced access to gender‐affirming surgeries and resources, increased mental health symptoms, and limited availability of in‐person therapy (Fish et al., 2020; Goldbach et al., 2020; Jarrett et al., 2021). Recent research has indicated that among LGBTQ emerging adults (18–23 years old) who lived with family during the COVID‐19 pandemic, watching LGBTQ YouTubers may moderate the links between family support, loneliness, and depression symptoms (Woznicki et al., 2020). Similarly, participants in this study actively engaged transvlogs to find trans‐created informational (69%), emotional (63%), and sociorelational (71%) resources. Furthermore, participants reported higher levels of self‐efficacy and well‐being than previously sampled trans and cisgender populations. Given this context, we suggest that therapists integrate transvlogs into their practice and support trans clients in their relational use of online resources and community. Transvlog therapeutic approaches encourage therapists to creatively engage trans affirmative clinical practices *within* extant community resource structures, thereby promoting client empowerment and agency.

Cinematherapy interventions provide a model for how to successfully integrate transvlogs into psychotherapy. Cinematherapy involves assigning clients movies to view between therapy sessions to expose clients to alternative coping strategies, facilitate therapeutic insights, and enhance client strengths (Berg‐Cross et al., 1990; Sharp et al., 2002). A prominent advantage of cinematherapy is that this method allows clients to explore and examine their treatment goals via an indirect, nonthreatening situation, which decreases the need to employ defense mechanisms (Schulenberg, 2003; Sharp et al., 2002). Furthermore, cinematherapy may be used to help clients identify resilience processes, as clients see others learn to survive and thrive amidst marginalized circumstances that mirror their own lives and identities (Carpenter et al., 2017; Eppler & Hutchings, 2020).

Therapists may enhance trans clients' resilience by assigning and discussing transvlogs addressing trans stigma. Therapists can use these transvlogs to validate their client's experiences while helping clients recognize their own strengths (Eppler & Hutchings, 2020). Therapists can reference Haas's (1995) three stages for integrating films into interventions when introducing transvlogs to clients: (a) preparation, in which the therapist assesses the client's suitability for this treatment method and selects a film based on the client's current treatment needs; (b) viewing, in which the client can identify with the characters and experience catharsis; and (c) discussion, in which the therapist assesses the client's identifications and new perspectives. Importantly, Eğeci and Gençöz (2017) have identified that the viewing itself does not promote change; rather, the discussion with the therapist afterward produces new insights into the client's problem areas. The therapist should begin the postviewing discussion with open‐ended questions about how clients responded to the transvlog and then follow‐up with more specific questions based on clients' feedback; this funnel‐approach to questioning enables clients to derive their own meaning from the transvlog under the therapist's guidance (Schulenberg, 2003).

For example, if a trans client is exploring their sexual orientation after beginning to take hormones, the therapist may assign ContraPoints' transvlog titled “Shame” (Wynn, 2020). Follow‐up discussion questions may include:

- What resonated for you about the vlogger's experience? Why?
- How did the vlogger explain their experience of shame? How did they work through those feelings?
- Were you surprised by the vlogger's sexuality experience after beginning to take hormones? Why or why not?
- How do you experience the intersection of gender identity and sexual orientation? What kinds of feelings have these identities evoked in you? What do you think accounts for that? How has your experience of those identities changed over time?
- Would you be interested in sharing this transvlog with others? If so, with whom? Why did you select this person? What do you hope this person will learn from the vlog?
- If you were to film your own transvlog, what would you focus on? Why?

The postviewing discussion provides clients with space to process reactions and feelings that may have arisen unexpectedly from the viewing (Heston & Kottman, 1997). By entering the world of the transvlogger with the client, the therapist forges a triadic relationship between therapist, client, and transvlogger (Heston & Kottman, 1997). Using Winnicott's conception of *potential space*, the transvlogger acts as a third plane that crosses the cultural divide between cisgender therapist and trans client (BenEzer, 2012). Thus, the transvlog becomes a *mutual creative space* in which the cisgender therapist and trans client cocreate a meaningful and effective therapeutic relationship (BenEzer, 2012). By identifying themselves in transvloggers, trans clients can gain insight into themselves and their relationships with others (Heston & Kottman, 1997). These relational insights create new opportunities for building trans resilience through processes such as mutual giving of support and community connectedness.

In addition, photovoice methodology exemplifies how images can be used to enhance trust and connection between therapist and client. Photovoice research suggests that images are a primary form of data that can convey meaning across languages and cultures (Christensen, 2018). Photovoice is a research method in which participants create and discuss photographs as a means of communicating their life experiences, expertise, and knowledge (Wang, 1999). Accordingly, photovoice research aims to empower underrepresented communities to articulate their perspectives by sharing and critically reflecting upon photographs, often of themselves (Christensen, 2018). Recent scholarship has explored photovoice research as a tool to empower trans patients to advocate for better care for themselves and communicate their healthcare needs to providers (Nelson, 2020). One study by Forge et al. (2018) utilized photovoice methodology to explore photographs as a means of empowering sexual and gender expansive youth to share their lived experiences of homelessness. Forge et al. (2018) found that discussing meaningful photographic images helped participants process their experiences, manage their inner struggles, and recognize their personal strengths. The results of these studies illustrate how transvlogs stand to facilitate trans clients' self‐reflection and therapeutic alliance.

The use of transvlogs as a self‐help supplement to psychotherapy is consistent with a strength‐based view of clients as active self‐healers and therapists as guides or facilitators (Bohart & Tallman, 1999; Prochaska, 1995). Within this framework, therapists provide an empathic workspace that promotes clients' innate capacity to heal and thrive (Bohart & Tallman, 1999). Given that trans clients often report negative experiences in psychotherapy (Mizock & Lundquist, 2016; Shipherd et al., 2010) and yet are often required to see therapists (e.g., 83% of the current sample had seen a therapist), we suggest that therapists expand their toolbox with the use of transvlogs. Therapists can meet American Psychological Association (2017) guidelines for trans affirmative therapy by utilizing transvlogs to build a positive rapport with trans clients that facilitates the introduction of advanced relational coping mechanisms. Transvlogs additionally provide an opportunity for therapists to educate themselves about trans issues and culturally appropriate communication techniques, rather than placing this burden on trans clients. Future research is warranted to explore the efficacy of transvlogs as a therapeutic tool for building relational resilience across the trans community.

### 4.4 Conclusion

The present mixed‐methods study contributes to a growing body of strength‐based trans research by exploring how trans individuals use transvlogs as resilience and community‐building tools. This study outlines how transvlog viewers actively utilized transvlogs as a means of gaining and giving resources in a relational exchange. Consequently, we argue that transvlogs can provide a dynamic platform for co‐constructing, navigating, and sustaining trans community as an essential component of resilience. However, given that more participants gained support than gave support to others, therapists may benefit from focusing on identifying and facilitating relational processes such as the mutual giving of support and community connectedness to empower trans clients.

The strengths of the current study include (a) using one of the largest mixed‐methods samples of transvlog viewers to date; (b) addressing trans‐specific resilience factors—something that is notably rare in the extant literature; and (c) applying a strength‐based perspective to explore the ways in which trans individuals connect to transvlogs to enact trans community resilience. Furthermore, this study breaks new ground by highlighting how the bidirectional process of sharing online resources can cultivate trans relational resilience and community. Finally, this study establishes a starting point for how therapists can utilize extant trans‐created resources to foster trans community resilience.

## CONFLICT OF INTERESTS

The authors declare that there are no conflict of interests.

## Footnotes

1 Trans is an umbrella term referring to individuals whose gender identity differs from the sex assigned to them at birth (American Psychological Association, 2015). We use the term trans rather than transgender to capture a broad myriad of genders within the trans community including those who have not taken steps to outwardly transition as well as those who identify outside of binary notions of gender (e.g., genderqueer or nonbinary).

2 Cisgender refers to those identifying with their sex assigned at birth (American Psychological Association, 2015).

3 Binding refers to a method of passing used by female‐to‐male trans individuals in which breasts are wrapped with household bandages or abdominal support garments to foster the impression of a masculine looking chest (Gauthier & Chaudoir, 2004).

4 One participant stated they did not watch transvlogs to gain any resources; they watched because “a friend of mine wanted to watch them.”

5 The fifth participant did not specify.

6 Gender was equally distributed between male and female participants. To our knowledge, there are no published trans reported means for this measure.
